# Bioenergetic shift and proteomic signature induced by lentiviral-transduction of GFP-based biosensors

**DOI:** 10.1016/j.redox.2024.103416

**Published:** 2024-11-02

**Authors:** Sarah Barakat, Şeyma Çimen, Seyed Mohammad Miri, Emre Vatandaşlar, Hayriye Ecem Yelkenci, Alejandro San Martín, Mustafa Çağlar Beker, Kıvanç Kök, Gürkan Öztürk, Emrah Eroglu

**Affiliations:** a Regenerative and Restorative Medicine Research Center (REMER), Research Institute for Health Sciences and Technologies (SABITA), Istanbul Medipol University, Istanbul, 34810, Turkey; b Department of Nutrition and Dietetics, Institution of Health Sciences, Istanbul Medipol University, Istanbul, 34810, Turkey; c Molecular Biology, Genetics, and Bioengineering Program, Faculty of Engineering and Natural Sciences, Sabanci University, Istanbul, 34956, Turkey; d Centro de Estudios Científicos (CECs), 5110466, Valdivia, Chile; e Facultad de Ciencias para el Cuidado de la Salud, Universidad San Sebastián, 5110773, Valdivia, Chile; f Department of Physiology, School of Medicine, Istanbul Medipol University, Istanbul, 34810, Turkey; g Department of Biostatistics and Medical Informatics, International School of Medicine, Istanbul Medipol University, Istanbul, 34810, Turkey; h Department of Physiology, School of Medicine, Bolu Abant İzzet Baysal University, Bolu, 14030, Turkey

**Keywords:** Green fluorescent proteins, HyPer7 genetically encoded biosensor, Hydrogen peroxide, Oxidative stress, Proteomic signature

## Abstract

Fluorescent proteins (FPs) stand as pivotal tools extensively employed across diverse biological research endeavors in various model systems. However, long-standing concerns surround their use due to the numerous side effects associated with their expression. Recent investigations have brought to light the significance of hydrogen peroxide (H_2_O_2_) that is associated with the maturation process of green fluorescent protein (GFP) fluorophores. The structural and functional impairments associated with GFP expression are possibly linked to this amount of H_2_O_2_. In this study, we assess the impact of the GFP-based HyPer7 biosensor on cellular homeostasis and proteome changes, aiming to identify potential risks related to oxidative stress responses that potentially risks the application of such tools. Cells expressing genome-integrated HyPer7 demonstrated altered mitochondrial membrane potential (MMP), which was alleviated by the addition of antioxidants or culturing cells at physiological normoxia (5 kPa O_2_). Additionally, HyPer7-expressing cells also exhibited significant impairment in mitochondrial oxidative respiration, suggesting broader mitochondrial dysfunction. Through untargeted proteomics analysis, we identified 26 proteins exhibiting differential expression in HyPer7-expressing cells compared to respective control cells. Functional annotation analysis showed that the list of the delineated proteins is associated with cellular responses to stress and the regulation of antioxidant mechanisms. Our findings underscore the significance of caution and validation in ensuring a thorough comprehension of cellular responses when using fluorescent protein-based tools, thereby enhancing the reliability of the results.

## 1 Introduction

Green fluorescent proteins (GFPs) are one of the most widely employed tools in biological research [1]. Nonetheless, their utilization is accompanied by considerable concerns raising from the various adverse effects linked to their expression, ranging from disruptions in cellular functions, to manifestations of cytotoxicity and immunogenic responses [[2], [3], [4]]. For example, proteomic profiling of GFP-expressing breast cancer cells revealed alterations in the expression levels of proteins linked to critical cellular functions, including protein folding, cytoskeletal organization, and cellular immune response [5]. These findings raise concerns about the relevance and interpretation of the results obtained by such GFP-based studies. Similarly, GFP-expressing prostate cancer showed attenuated tumorigenicity, which was attributed to their altered cellular energy metabolism and cytoskeletal dynamics [6]. Furthermore, in animal models, the introduction of various FPs, whether through transgenic approaches or lentiviral vector-mediated delivery, has been shown to induce numerous adverse effects on critical cardiac function [[7], [8], [9]].

Despite the clear detrimental effects of GFP expression on cells, the detailed molecular mechanisms underlying these effects remain not fully understood. Recent investigations have highlighted the significance of H_2_O_2_ produced during the GFP maturation process within the cellular environment [[10], [11], [12]]. This H_2_O_2_ amount was usually neglected given the perception that it fell within the cellular baseline levels, and therefore, not expected to induce any toxicity or have any major effect on the cellular homeostasis [13]. However, recent studies show that this amount surpasses typical physiological levels, potentially being responsible for the observed structural and functional deteriorations [14]. This is comprehensible, as H_2_O_2_ is a key reactive oxygen species (ROS) involved in numerous signaling pathways [15]. Consequently, any dysregulation in its precise control can be of major significance and potentially have a key role in the onset of an array of oxidative stress related conditions [16,17]. In this context, Ganini et al. in their transcriptomics study, demonstrated a correlation between the increased levels of H_2_O_2_ generated during enhanced GFP (eGFP) maturation and the potential activation of the HIF1α pathway [12]. Furthermore, Goto et al. reported that eGFP elevates oxidative stress, concomitant with a significant increase in the major antioxidant molecule, glutathione [18]. Stress-related genes were also found to be upregulated in mouse neuronal cells expressing yellow fluorescent protein (YFP) [19].

The GFP-based biosensor, HyPer7, stands out as a potent tool for precisely monitoring H_2_O_2_ in living cells [20,21]. Given the association between GFP maturation and the production of H_2_O_2_, it has prompted us to pose a significant question: *Does the utilization of GFP-based biosensors, such as HyPer7, possibly induce oxidative stress itself within the cellular milieu, consequently impacting overall cellular homeostasis?* In this study, we provide insights to address these questions. We evaluated the mitochondrial functions in endothelial and keratinocyte cells stably expressing the HyPer7 biosensor, recognizing mitochondria as the primary source of ROS regulation in cells [22]. Subsequently, we examine the proteomic profiles of endothelial cells and unveil their potential association with oxidative stress related pathways. This study addresses the pressing need for a thorough reevaluation of these tools in their commonly applied methodologies.

## 2 Materials and methods

### 2.1 Stable cell line generation

Endothelial cells (EA.hy926: ATCC, CRL-2922, Manassas, VA, USA) and primary epidermal keratinocytes (HEKa: ATCC, PCS-200-011, Manassas, VA, USA), stably expressing the HyPer7 biosensor, were generated as explained previously [20]. In brief, cells were cultured in DMEM (ATCC, Manassas, VA, USA) supplemented with 10 % FBS, 100 μg/mL Penicillin, 100 U/mL Streptomycin, 100 μg/mL Normocin (InvivoGen, San Diego, CA, USA), and 2 % HAT (Sodium Hypoxanthine (5 mM), Aminopterin (20 μM), and Thymidine (0.8 mM)) (ATCC, Manassas, VA, USA) for EA.hy926. The cells were maintained in a humidified CO_2_ incubator (5 % CO_2_, 37 °C), at standard (18 kPa) O₂ or adapted to (5 kPa) O₂ [23]. For viral transduction, the cells were seeded on a 6-well plate in an antibiotic-free transduction medium containing 10 % FBS, 10 μg/mL Polybrene infection reagent (Sigma-Aldrich, St. Louis, MO, USA), and lentivirus particles encoding for the cytosolically targeted HyPer7. The cells were cultured in the virus-containing medium for 48–72 h. Subsequently, the cells were cultured for an additional week in complete DMEM before undergoing fluorescence-activated cell sorting (FACS) using a B.D. Influx Cell Sorter with appropriate settings (488 nm laser, 530/40 nm band-pass filter). The cell population exhibiting positive green fluorescence was gated from the negative population and sorted. This selected pool of cells was then further cultured and utilized in subsequent experiments.

### 2.2 Measurement of mitochondrial membrane potential using TMRM

Mitochondrial membrane potential (Δψm) was measured using tetramethylrhodamine methyl ester (ab113852, Abcam) following the manufacturer's instructions. Wild-type and HyPer7-expressing cells at the same passage number were grown on 30 mm glass coverslips (Glaswarenfabrik Karl Knecht Sondheim, Sondheim vor der Rhön, Germany) to 80 % confluency. For vitamin C treatment, cells were incubated in 100 μM vitamin C for 24 h prior to staining, with the medium refreshed every 6 h. On the experiment day, the medium was replaced with fresh medium containing 20 nM TMRM, and cells were incubated for 30 min at 37 °C in the dark. After incubation, cells were washed with PBS and maintained in a storage buffer until cells were imaged in HEPES-buffered solution [20], with a pH of 7.42, using a Zeiss Axio Observer.Z1/7 inverted widefield epifluorescence microscope (Carl Zeiss AG, Oberkochen, Germany) with a Plan-Apochromat 20 × /0.8 dry objective. TMRM fluorescence was excited at 555/30 nm and detected with a 605/70 nm emission filter. For HyPer7-expressing cells, fluorescence signals were alternately excited at 420 nm and 490 nm, with emissions collected using a BP 525/50 bandpass filter. Data were acquired using Zen Blue 3.1 Pro software.

### 2.3 Analysis of cellular metabolism

Cellular oxygen consumption rate (OCR) and extracellular acidification rate (ECAR) of both wild-type and HyPer7-expressing cells were measured with a Seahorse XFe96 Extracellular Flux Analyzer (Agilent Technologies). The day before measurement, cells were seeded at a density of 1.2 × 10^4^ and 2 × 10^4^ cells per well for EA.hy926 and HEKa cells, respectively, on Seahorse XFe96 plates and incubated for 24 h. On the day of analysis, the cell culture medium was replaced with a base medium supplemented with 10 mmol/L glucose, 1 mmol/L sodium pyruvate, and 2 mmol/L l-glutamine for the Mito Stress Test, and with a base medium supplemented with 2 mmol/L l-glutamine for the Glycolysis Stress Test. Cells were then incubated in a 37 °C incubator without CO_2_ for 1 h. All media and injection reagents were adjusted to pH 7.4 on the day of the assay. For the Mito Stress Test, OCR was analyzed under basal conditions and after sequential injections of 2 μmol/L oligomycin, 1.5 μmol/L carbonyl cyanide-4 (trifluoromethoxy) phenylhydrazone (FCCP), and 1 μmol/L rotenone and antimycin A. In the Glycolysis Stress Test, after basal measurements, glucose, oligomycin, and 2-deoxyglucose (2-DG) were subsequently injected into the medium to final concentrations of 10 mmol/L, 1 μmol/L, and 50 mmol/L, respectively. The analysis of the results was conducted using Wave 2.6 software (Agilent Technologies). Data obtained for each condition were normalized to the cell number using DAPI counts of each well and presented as pmol/min/10^4^ cells for OCR and mpH/min/10^4^ cells for ECAR.

### 2.4 Comparative proteomic profiling

#### 2.4.1 Experimental Design of the proteomics experiment

Comparative proteomic profiling was performed by comparing a control group with an experimental group of 4 biological replicates for each. The control group represented wild-type EA.hy926 cells, while the experimental group represented EA.hy926 cells stably expressing the HyPer7 biosensor in the cytosol.

#### 2.4.2 Sample preparation for liquid chromatography mass spectrometry (LC-MS)

Proteins were extracted using a protein extraction kit (UPX Universal; Expedon). Cells were resuspended in Tris-HCl with a protease inhibitor, then sonicated for 10 min. The mixture was then added to UPX buffer in a 1:1 ratio before being heated at 100 °C for 10 min. The samples were then incubated at 4 °C for 1 h, followed by centrifugation at 14,000 g for 20 min. The supernatants were then collected, and protein concentration was determined using the Qubit 3.0 Fluorometer according to the manufacturer's instructions (Q33216, Invitrogen, Life Technologies Corporation, Carlsbad, CA, USA). Protein digestion was performed using the FASP protein digestion kit (ab270519, Abcam, Cambridge, UK) following the standard protocol as described elsewhere [24]. Herein, 50 μg of protein samples were filtered using a 30 kDa cut-off spin column in 6 M urea. Subsequently, the samples are alkylated with 10 mM iodoacetamide at room temperature for 20 min in the dark. The samples were finally incubated at a 1:100 ratio with MS grade trypsin protease (90,057, Thermo Fisher Scientific, Waltham, MA, USA) overnight at 37 °C. The next day, peptides were eluted from the columns and lyophilized. The peptides were suspended in 0.1 % formic acid (1,002,642,510, Merck) and diluted to a final concentration of 100 ng/μL before being supplied to the LC-MS system.

#### 2.4.3 Liquid chromatography mass spectrometry analysis

The ACQUITY UPLC M-Class, coupled to a SYNAPT G2-Si high-definition mass spectrometer (Waters, Milford, MA, USA), was employed following optimized methods for cells untargeted proteomics analysis. The column was equilibrated with 97 % mobile phase A (0.1 % FA UHPLC grade water), and the column temperature was set to 55 °C. Subsequently, samples were eluted from the trap column (ACQUITY UPLC M-Class Symmetry C18 trap column, 180 μm × 20 mm; 186007496, Waters) to the analytic column (ACQUITY UPLC M-Class HSS T3 Column, 100 Å, 1.8 μm, 75 μm × 250 mm; 186007474, Waters) using a 90 min gradient elution setup of mobile phase B (hypergrade acetonitrile containing 0.1 % FA, v/v) from 4 % to 40 % at a flow rate of 0.400 μL/min. The resolution mode in positive ionization was employed with a 0.6-s cycle time. Data-independent acquisition mode was utilized with an *m*/*z* range of 50–1900. For mass calibration, 100 fmol/μL of Glu-1-fibrinopeptide B was applied in a 45-s interval.

### 2.5 Data analysis

#### 2.5.1 Statistical analysis of bioenergy experiments

The obtained numerical data from the Seahorse and microscopic experiments were summarized using the mean ± s.e.m (as descriptive statistics) of at least three independent measurements. Statistical significance was computed by applying a two-tailed independent sample Student's t-test. Statistical analyses were performed using GraphPad Prism (Version 10.1.1, San Diego, CA). P < 0.05 was considered statistically significant. Other statistical specifications are indicated where relevant.

#### 2.5.2 Mass spectrometry data analysis

##### 2.5.2.1 Mass spectrometry raw data analysis

Peptide identification and quantification were conducted using Progenesis-QI software (Waters) in conjunction with the Uniprot Human database, accessed on September 12, 2023 [25]. Various parameters were configured, including a low energy threshold of 200 counts, a high energy threshold of 50 counts, and an intensity threshold of 750 counts. Wild-type cells (control samples) were selected to establish a reference for alignment. The "normalize to all peptide ions" option was utilized for normalization, wherein the software identifies the sample that exhibits minimal difference from the others and calculates a normalization factor for the other samples based on the mean of the log abundance ratios of each ion that falls within the robust estimated limits. Peptide ions beyond these limits were deemed outliers and were not included in the calculation of the normalization factor. Additional settings included a maximum allowed charge of 5, a false discovery rate (FDR) of <4 %, allowance for one missed cleavage, fixed carbamidomethylation, variable methionine oxidation, a maximum protein mass of 400 KDa, and ion matching requirements of ≥3, ≥7, and ≥1 for fragments per peptide, fragments per protein, and peptides per protein, respectively. A minimum of 1 unique peptide was employed for whole protein identification, followed by the calculation of protein expression ratios. Consequently, a list of identified proteins with their respective abundance levels was generated for subsequent analysis.

##### 2.5.2.2 Mass spectrometry downstream data analysis

a) Differential Expression Analysis and Multivariate Statistical Analysis:

The differential expression analysis consisted of two parts: univariate statistical analysis and multivariate statistical analysis. In the univariate statistical analysis, each protein is treated as an individual variable. Differences between the control and experimental groups were evaluated statistically using independent samples. Student's t-test on the normalized protein expression data of Progenesis-QI software was employed. Proteins with (p < 0.05) were considered as significantly deferentially expressed proteins (DEPs). The top differentially expressed proteins (|fold change| ≥1) were considered as top DEPs, and these proteins constituted the input list for subsequent functional annotation analysis and protein-protein interaction (PPI) network analysis. Multivariate statistical analysis consisted of hierarchical clustering analysis (HCA) and principal component analysis (PCA). The input files for these analyses consist of the list of investigated proteins along with their relative expression levels. Hierarchical clustering analysis was performed using SRplot web tool [26] (accessed on January 13, 2024), where scaling was applied to rows, cluster orientation was bidirectional, Pearson correlation was used for the distance metric, and average linkage was applied as linkage method. The PCA was performed using ClustVis 0.2 web tool (BETA version-large data edition, accessed on January 13, 2024) [27], using unit variance scaling to rows and singular value decomposition (SVD) to calculate principal components (PCs). The PCA plot was generated using the first two PCs (the two major PCs) and accordingly showed separately the corresponding explained variances in the respective axis titles. To obtain complementary findings on sample grouping based on the expression of top DEP, a PCA biplot was generated using Jamovi software (Version 2.3.28) [28].

b) Functional Annotation Analysis (FAA):

The Database for Annotation, Visualization, and Integrated Discovery (DAVID) (accessed on October 5, 2024) was used for FAA [29,30]. The list of significantly top DEPs was used as input, with the total detected proteins serving as the background. The count threshold and EASE score were kept at the default values (2 and 0.1, respectively). The analysis was based on the following functional annotation databases: the three parts of gene ontology (namely: biological processes (BP), cellular components (CC), and molecular functions (MF)), REACTOME Pathway, and UCSC TFBS. For each analysis, a list of the top 10 significant enriched terms (arranged in descending order according to their p-values) was identified and prioritized. These “top 10” enriched terms were displayed in a dot plot in relation to the number of proteins (count), p-value, and enrichment value using the SRplot tool.

c) Protein-Protein Interactions, Network Analysis, and Pathway Mapping:

The String database (version 12.0, accessed on January 21, 2024, RRID:SCR_005223) was used to retrieve the available protein-protein interactions (PPI) among the top DEPs [31]. For the retrieval, the minimum required interaction score was selected as ‘medium confidence:0.400’, the meaning of network edges was selected as ‘evidence’, all options for active interaction source were selected, and the maximum number of interactors was selected as ‘none-query proteins only’. Clustering for this PPI network was performed using the String k-means clustering method. The obtained PPI network underwent functional annotation analysis using the String database before being imported into Cytoscape software (version 3.10.1) [32] for further network analysis and visualization.

Pathway maps were generated using the SRplot tool for the tricarboxylic acid cycle (TCA cycle), oxidative phosphorylation, and glycolysis/gluconeogenesis pathways. The KEGG pathway IDs of the forementioned pathways, protein entrez IDs of the DEPs deferentially expressed proteins, and fold change of each protein were used as inputs. Biorender software was then used to generate simplified, representative figures of these pathways for ease of visualization. The figures incorporate a relative color scale indicating the fold change of the involved proteins.

### 2.6 Data availability

The mass spectrometry proteomics raw data and the normalized protein expression dataset have been deposited to the jPOST repository with the ID JPST003409 [33] and with the ProteomeXchange ID PXD056727. The data can be accessed through the following links: jPOST [https://repository.jpostdb.org/entry/JPST003409] and ProteomeXchange [https://proteomecentral.proteomexchange.org/cgi/GetDataset?ID=PXD056727].

## 3 Results and discussion

In this study, we aimed to assess the impact of HyPer7 biosensor expression, as a GFP-based biosensor on general cellular homeostasis, potentially related to the disruption of ROS equilibrium. We used an immortalized human umbilical vein endothelial cell line (EA.hy926) stably expressing the cytosolic HyPer7 biosensor. Endothelial cells possess intricate mechanisms to regulate oxidative stress. Even subtle changes in ROS levels within these cells may potentially contribute to various dysfunctions and vascular pathologies [[34], [35], [36]], thus this cell line was established in this study as a model system. Additionally, we used HEKa keratinocytes, which are more resilient to oxidative stress, to confirm our results across different cell types.

### 3.1 HyPer7 expression impacts MMP and cellular energy metabolism

**Fig. 1:**
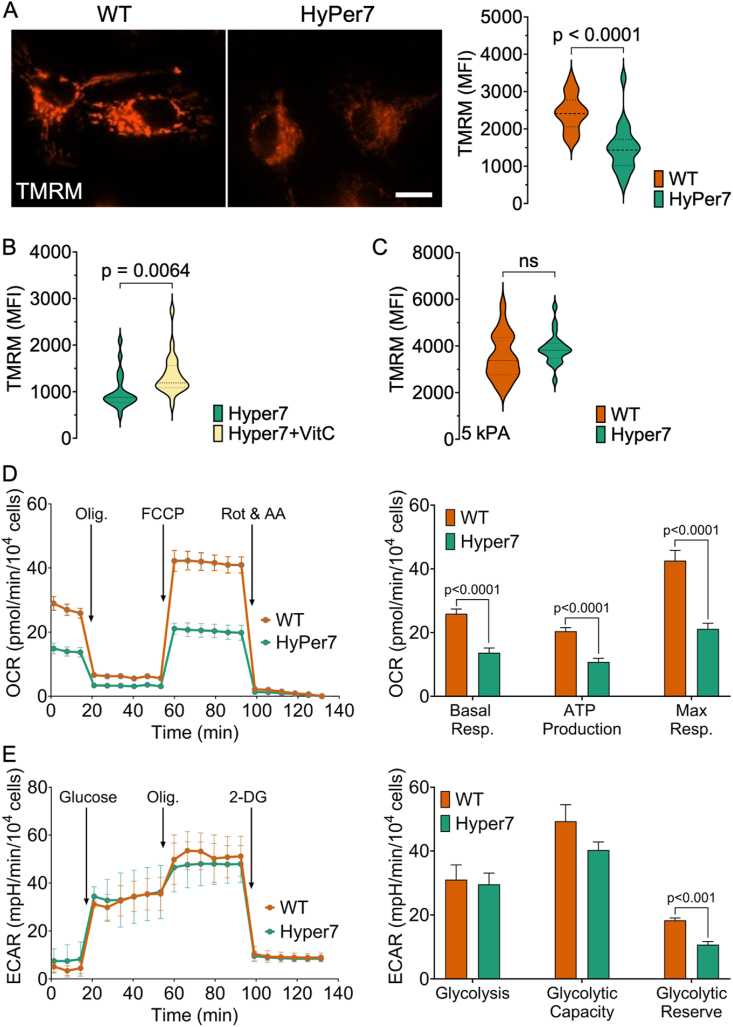
**A)** Microscopic analyses of mitochondrial membrane potential (Δψm). Left panel: representative images of wild-type EA.hy926 cells (left) and HyPer7-expressing cells (right) stained with 20 nM of TMRM. Right panel: violin plots of quantitative analysis of TMRM fluorescence in wild-type cells (orange) in comparison to HyPer7-expressing cells (green). **B)** Violin plots of quantitative analysis of TMRM fluorescence in wild-type EA.hy926 cells (orange) in comparison to HyPer7-expressing cells (green) after 24 h incubation in 100 μM of vitamin C. **C)** Violin plots of quantitative analysis of TMRM fluorescence in wild-type EA.hy926 cells (orange) in comparison to HyPer7-expressing cells (green) adapted to 5 kPa. (n = ∼100). **D)** Line plot showing comparison of oxygen consumption rate (OCR), **E)** extracellular acidification rate (ECAR) of HyPer7-expressing cells (green) in comparison to wild-type cells (orange). The data are representative of three independent experiments with n = 3 for each cell line. Data are expressed as mean ± s.e.m.

Mitochondria are highly impacted by fluctuations in cellular ROS levels [34]. Thus, we initially investigated the effect of HyPer7 expression on mitochondrial function. TMRM staining of EA.hy926 cells revealed a significant decrease in the MMP (Δψm) in HyPer7-expressing cells compared to their wild-type counterparts (Fig. 1A, Supp Fi g. S1 A). We observed the same effect in HEKa cells, further confirming the results (Supp F ig. S1.C). To assess whether these results were related to H₂O₂, we incubated the cells with vitamin C prior to TMRM staining. Indeed, vitamin C alleviated the effect on MMP in HyPer7-expressing cells, suggesting a role for oxidative stress in the observed changes (Fig. 1B, Supp F ig. S1 C).

Given the influence of oxygen levels on cellular redox states, we aimed to address this effect on our results. Both we and others have shown that cells adapted to physiological normoxia (5 kPa O₂) exhibit lower levels of Nrf2 and its target genes, indicating a more active antioxidant machinery under these conditions [23,37,38]. We found that HyPer7-expressing cells adapted to 5 kPa O₂ showed no significant difference in MMP compared to wild-type cells, likely due to the enhanced antioxidant machinery at physioxic oxygen levels (Fig. 1C, Supp F ig. S1 B,D).

To investigate whether these results were influenced by GFP expression levels, we hypothesized that higher HyPer7 expression would correlate with a stronger effect on TMRM staining. Therefore, we performed a correlation analysis between HyPer7 expression levels and TMRM staining, considering that stable cell lines can be heterogeneous in expression. Our analysis showed a range of HyPer7 expression levels across the EA.hy926 and HEKa cell lines; however, no significant correlation was found between HyPer7 expression and TMRM staining (Supp F ig. S1 E). This analysis suggests that even low levels of HyPer7 expression significantly affect the MMP.

These findings suggest that the expression of HyPer7 under conventional culture conditions may lead to changes in oxidative respiration. Therefore, we next examined the impact of HyPer7 expression on cellular energy metabolism utilizing the Seahorse XF Analyzer. We quantified the OCR and the ECAR as indicators of oxidative respiration and glycolysis, respectively. The basal OCR in cells expressing HyPer7 exhibited a significant reduction compared to wild-type cells, in accordance with TMRM staining results, indicating diminished mitochondrial respiration under baseline conditions (Fig. 1D, Supp. Fig. S1. F). Moreover, both ATP production capacity and maximal respiration were compromised in these cells (Fig. 1D, Supp F ig. S1.F). The observed reduction in OCR could be conceivably linked to the impact of increased oxidative stress within these cells, potentially resulting in mitochondrial impairment and dysfunction [39,40]. In response to this impairment, cells may exhibit an upregulation of glycolysis as an adaptive mechanism to sustain cellular energy homeostasis and attenuate ROS production [41,42]. However, analysis of the ECAR, indicated no significant alterations between the two cell types at both basal and maximum levels (Fig. 1E, Supp. Fig. S1, F). However, the glycolytic stress test experiments were conducted with cells preincubated with l-glutamine. Under such conditions, cells are likely to undergo metabolic reprogramming toward oxidative metabolism [43]. This shift could explain the lack of glycolytic activation observed in HyPer7-expressing cells.

It is noteworthy that the Seahorse analysis and TMRM staining experiments were conducted using two distinct cell lines (EA.hy926, HEKa) expressing the HyPer7 biosensor through separate rounds of virus transduction and stable cell line generation. Consequently, the results presented herein are less likely to be attributed to defects at sites of plasmid integration within the genome [44]. These findings suggest that HyPer7 expression influences cellular energy metabolism, particularly mitochondrial respiration, which may contribute to the disruptions in cellular homeostasis.

### 3.2 HyPer7 expression substantially alters cellular proteomic profile

**Fig. 2:**
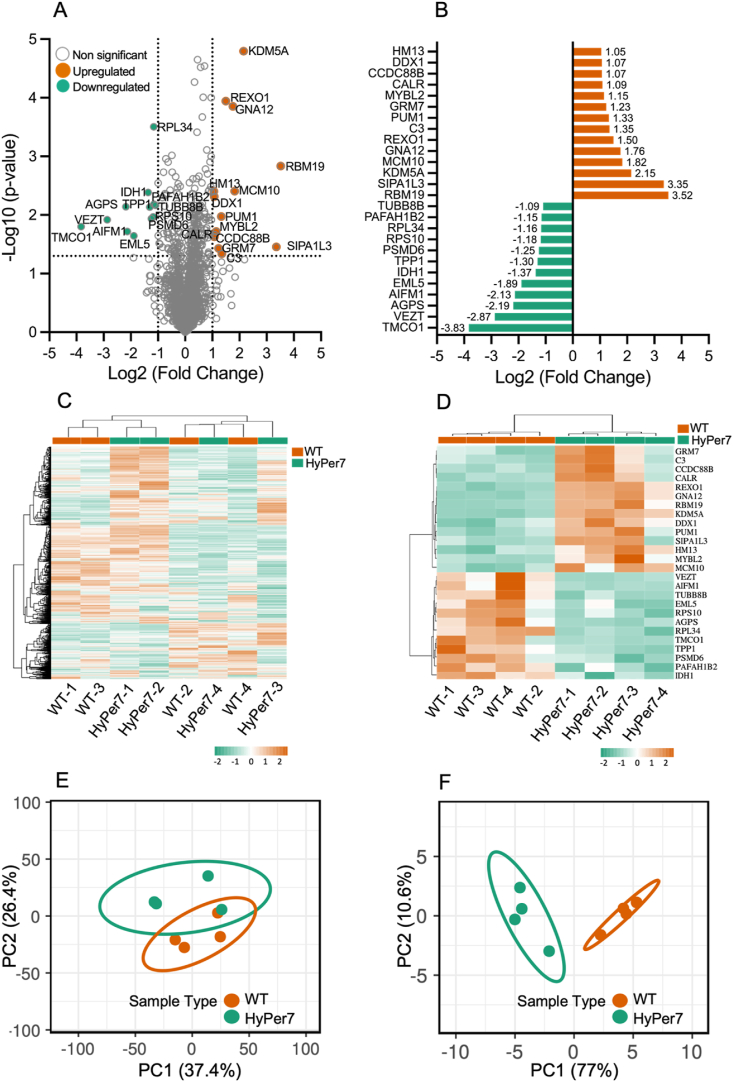
**A)** Volcano plot illustrating all identified proteins (1662) by LC-MS analysis in HyPer7-expressing cells in comparison to wild-type cells. Upregulated top DEPs (14) and downregulated (12) are highlighted in orange and green, respectively, while non significantly expressed proteins are shown in grey. **B)** List of top DEPs in HyPer7-expressing cells compared to wild-type cells, with their respective Log2 fold change values. Green bars: significantly downregulated proteins, orange bars: significantly upregulated proteins. **C, D)** Hierarchical clustering heatmap analysis for total identified proteins and for top DEPs, respectively. The profile highlights the clustering of samples according to their sample type: HyPer7 compared to control wild-type cells. The orange and green color scale shows upregulated and downregulated protein expression, respectively. Scaling was applied to rows, cluster orientation was bidirectional, Pearson correlation was used for the distance metric, and average linkage was applied as the linkage method. **E, F)** PCA for total identified proteins and for top DEPs, respectively. Graphs display separation of the two sample groups, HyPer7 (green) compared to control wild-type cells (orange), based on the protein expression. The X and Y axes show principal component 1 and principal component 2, respectively. Prediction ellipses are such that, with a probability of 0.95, a new observation from the same group will fall inside the ellipse.

Observing changes in the energy metabolism of HyPer7-expressing cells, we next aimed to examine their proteomic profiles, checking for affected pathways and determining if oxidative stress-related pathways were impacted. The proteomics workflow is shown in (Supp. Fig. S2). Untargeted proteomics analysis of wild-type and HyPer7-expressing cells resulted in the identification of 1662 proteins. The applied Student's independent *t*-test revealed a list of 250 DEPs (Supp F ig. S3 A, B). As a complementary step, the log2 fold change of these proteins was calculated, resulting in the identification of 26 top DEPs (14 upregulated and 12 downregulated) (Fig. 2 A, B). The resulting list of top DEPs, including the related official protein symbol and full protein names, along with the statistical details, is provided in Supp. Table S1.

Hierarchical clustering analysis (HCA) was independently conducted for both sets of proteins; the entire set of identified proteins and for the 26 top DEPs. The dendrogram of hierarchical clustering of samples based on the expression of the top DEPs demonstrated grouping of samples according to their sample group (wild-type vs. HyPer7-expressing cells), while when considering the entire set of identified proteins, the clustering method failed to effectively segregate the two distinct sample types (Fig. 2 C, D). These results suggest that the expression of HyPer7 did not have a profound impact on the overall cellular proteome; rather, its effect was predominantly confined to a subset of proteins. Consistent with the HCA results, the PCA plots generated for top DEPs revealed distinct clustering of the examined samples according to the sample group (wild-type vs. HyPer7-expressing cells) in comparison to what was observed in the case of the complete set of identified proteins (Fig. 2 E, F). Further insights were obtained into the top DEPs in the context of the separation of sample groups using the PCA biplot (Supp F ig. S3 C, D). In conclusion, the clear separation patterns observed in both the HCA plot, and the PCA plots based on the expression of top DEPs collectively underscore the indicative relevance of these proteins in distinguishing the two sample groups.

### 3.3 HyPer7 expression enriches stress related pathways

**Fig. 3:**
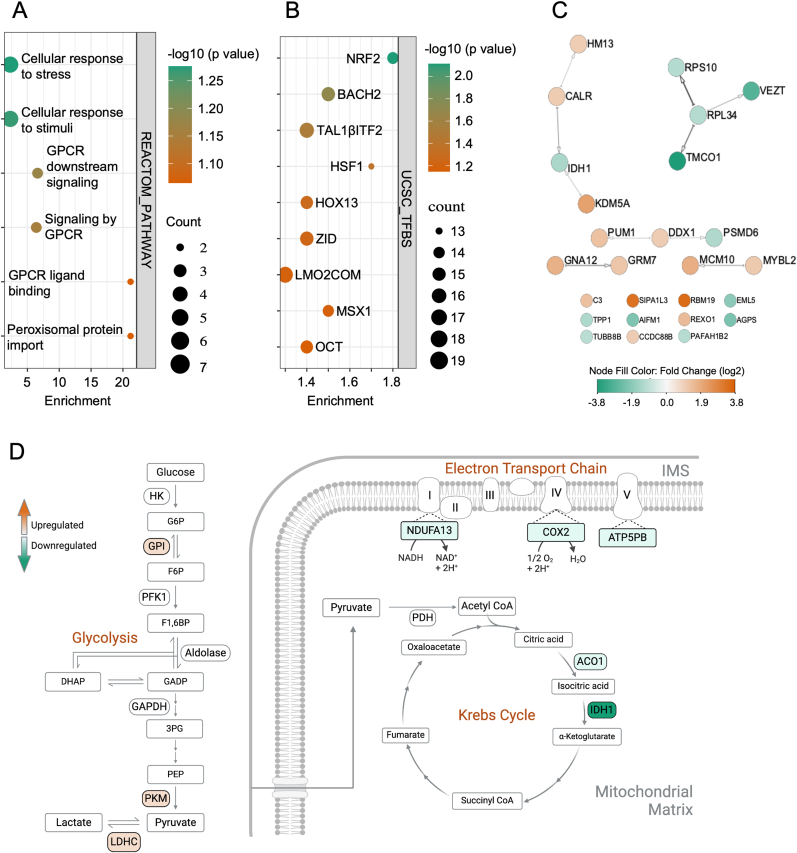
**A, B)** Dot plot of Functional enrichment analysis of REACTOME PATHWAYS, and UCSC TFBS enriched terms for the top DEPs. Circle size represents the number of proteins enriched in each term, while the color of the circles represents the significance of this enrichment. **C)** Protein-protein interaction network (PPI) analysis for the top DEPs in HyPer7-expressing cells compared to wild-type cells. Nodes represent proteins, and edges represent protein-protein interactions (identified by the String database), node color represents log2 fold change; up-regulation (orange), down-regulation (green). The proteins with less than three connections are listed at the bottom of the graph. **D)** Schematic of pathway mapping illustrating changes in protein expression levels associated with glycolysis, Krebs cycle, and the electron transfer chain (ETC) in HyPer7 expressing cells compared to wild-type cells. The color bar gradient in the form of arrows represents log2 fold change, wherein: orange and green show upregulation or downregulation respectively.

Next, our aim was to evaluate the impact of the proteins exhibiting significant expression and elucidate the pathways in which they are implicated. Functional annotation analysis of the top DEPs using the REACTOME database revealed 6 enriched terms (Supp. Table S2). The term “cellular responses to stress” was the first enriched term (Fig. 3. A). Cellular responses to stress are a group of cellular processes and mechanisms that are activated in response to various types of stressors, including oxidative stress. Identified proteins in relation to cellular responses to stress included two upregulated proteins (CALR, HM13), and five downregulated proteins (IDH1, RPL34, RPS10, TPP1, PSMD6) (Supp. Table S1). Interestingly, CALR, an endoplasmic reticulum chaperone pivotal for regulating protein folding and calcium balance, has been observed to undergo upregulation in response to oxidative stress, consequently triggering apoptosis [45,46]. RPL34 and RPS10, on the other hand, are ribosomal proteins reported to be downregulated in cellular responses to oxidative stress [47]. These results suggest that HyPer7 expression may impact the regulation of oxidative stress-related genes.

The transcription factor binding site (TFBS) analysis by UCSC-TFBS database revealed the enrichment of binding sites for a list of transcription factors related to oxidative stress, and the top enriched transcription factor was NRF2 (Fig. 3. B). This transcription factor plays a major regulatory role in the antioxidant machinery by inducing the expression of a variety of genes involved in detoxification, antioxidant defense, and cellular protection against oxidative damage [48]. Identified proteins with enriched binding sites of NRF2 included 15 proteins: AGPS, AIFM1, DDX1, EML5, GNA12, GRM7, HM13, KDM5A, PAFAH1B2, PSMD6, TMCO1, CALR, RPL34, TPP1, and VEZT (Supp. Table S1). Enrichment of binding sites of this transcription factor may suggest a possible association of these proteins with antioxidant responses. Interestingly, the list of enriched transcription factors also included: BTB and CNC Homology 2 (BACH2), which was found to promote apoptosis under oxidative stress [49,50], and Heat Shock Factor 1 (HSF1) which is a ROS activated transcription factor that coordinates with NRF2 in orchestrating antioxidant defense mechanisms and preserving cellular homeostasis [51]. Additionally, proteins such as CALR have been associated with ER stress responses induced by unfolded proteins [52]. Gene ontology analysis did not reveal any significantly enriched terms in relation to biological processes (BP). Enriched terms related to cellular components (CC) and molecular functions (MF) are shown in (Supp F ig. S4 A).

We proceeded to investigate the PPI of the top DEPs in HyPer7-expressing cells using the STRING database. The resultant PPI network exhibited 26 nodes/proteins connected by 10 edges/interactions (Fig. 3. C). Notably, the average local clustering coefficient was calculated to be 0.423, accompanied by a PPI enrichment p-value of 0.139. Despite the non-significant p-value, potentially attributable to the relatively small number of input proteins, our analysis of the REACTOME database annotations associated with this protein network revealed "cellular responses to stress" as the top enriched term. The number of associated proteins with this term was 8, including: TPP1, CALR, RPL34, HM13, IDH1, PSMD6, RPS10, and TUBB8B. This signifies the association between our list of top DEPs and proteins implicated in cellular stress responses.

Given the fine-tuned regulation of H_2_O_2_ in cells [53], we sought to explore the PPI network of the DEPs. As some proteins may lack significant fold changes, it is still noteworthy to consider their biological relevance. The resultant PPI network comprised 248 nodes interconnected by 791 edges. The average local clustering coefficient was 0.424, with a PPI enrichment p-value of <1.0e-16 (Supp F ig. S5 A). Once again, "cellular responses to stress" emerged as the top enriched term (FDR = 5.49 e-13) in REACTOME Pathway analysis associated with this network of proteins. The number of proteins annotated with this term in our DEPs network was 43 of total the 747 proteins assigned to this term. In order to get more insight into these proteins and the types of stress they are associated with, we next employed the String clustering method to identify available clusters among our 43 stress-related proteins. This method utilizes the calculated distance matrix derived from the string global scores to identify available clusters. The clustering results identified 3 distinct clusters (Supp F ig. S5 B). Interestingly, one cluster consisted of 5 proteins: peroxiredoxin1 (PRDX1), peroxiredoxin3 (PRDX3), Sulfiredoxin-1 (SRXN1), NAD(P)H dehydrogenase (quinone) 1 (NQO1), and glutamate-cysteine ligase modifier subunit (GCLM). Functional annotation analysis of this cluster of proteins revealed enrichment of many terms related to cellular response to oxidative stress in relation to BPs. MFs enriched terms were mainly associated with antioxidant activity, namely thioredoxins. Moreover, NFE2L2 related pathways were enriched in both REACTOME Pathway and WikiPathways analysis. Details of enriched terms in association with BPs, MFs, REACTOME Pathway, and WikiPathways are given in Supp F ig. S4 B. Interestingly, upregulation of such oxidative stress related proteins in association with GFP expression was reported by Yanar et al. in their study on prostate cancer cells, wherein they reported upregulation of PRDX6 [6].

Based on our observation of altered MMP and impaired oxidative respiration through Seahorse and TMRM analyses, we aimed to map significantly differentiated proteins onto TCA cycle, oxidative phosphorylation, and glycolysis/gluconeogenesis pathway maps using the KEGG pathway mapping tool. With the small number of our top DEPs, which is not informative for such analysis, we decided to use a list of DEPs. Our pathway mapping unveiled a reduction in a pivotal TCA cycle component, isocitrate dehydrogenase 1 (IDH1) (log2 FC = −1.3). This significant downregulation could potentially elucidate the impaired mitochondrial respiration observed in HyPer7-expressing cells in seahorse analysis (Fig. 3. D). The pathway mapping also detected downregulation of a second TCA cycle contributor, aconitase 1 (Aco1) (log2 FC = −0.61). Moreover, certain proteins involved in mitochondrial electron transport chain (ETC) structure formation were found to be downregulated. These include subunits of the ATP synthase complex (ATP5PB), also referred to as Complex V, Cytochrome *c* Oxidase Subunit 2 (COX2) from Complex IV, and Ubiquinone Oxidoreductase Subunit A13 (NDUFA13) of Complex I (log2 FC = −0.24, −0.34, and −0.48, respectively) (Fig. 3D). These findings further corroborate the impairment of oxidative respiration in these cells.

Cells experiencing oxidative stress may exhibit compromised energy metabolism, favoring a shift towards glycolysis [39]. Although Seahorse analysis did not detect compensatory glycolytic pathway activation due to previously discussed experimental conditions, pathway mapping revealed upregulation of pivotal glycolytic enzymes, including glucose-6-phosphate isomerase (GPI), pyruvate kinase M (PKM), and lactate dehydrogenase C (LDHC) (log2 FC = 0.09, 0.23, and 0.85, respectively) (Fig. 3D). Therefore, the pyruvate generated within these cells is more likely to be converted to lactate rather than entering the impaired TCA cycle. These observations align with previous reports of enhanced glycolysis linked to GFP expression in prostate cancer cells [6]. Specifically, PKM upregulation, along with five other glycolysis-associated proteins, were reported [6], corroborating our findings. This data might suggest compensation for the impaired mitochondrial respiration in these cells through the activation of glycolytic pathways to meet energy demands.

## 4 Conclusions and suggestions for future studies

Among other H_2_O_2_ probes, HyPer7 biosensor stands out as one of the most efficient methods, due to its exceptional sensitivity in precise spatiotemporal detection of H_2_O_2_ [21]. In this study, we addressed the impact of stable HyPer7 expression on general cellular homeostasis, considering the recognized association between GFP maturation and H_2_O_2_ production [12,14].

Our findings suggest the presence of heightened oxidative stress levels in the cells, as indicated by the enrichment of stress-responsive pathways and the binding sites of NRF2. Moreover, our data indicate a metabolic dysfunction in these cells characterized by impaired mitochondrial respiration and suggest compensatory upregulation of glycolysis. These metabolic alterations might be due to generated H_2_O_2_ during GFP maturation or possibly induced by heterologous protein expression itself. While previous studies have documented the generation of H₂O₂ during GFP maturation, further investigation is required to determine the exact contribution of this process versus heterologous protein overexpression to homeostasis perturbation. Future studies should focus on comparing basal H₂O₂ levels in GFP-expressing cells with their wild-type counterparts. However, quantifying basal intracellular H₂O₂ presents challenges due to the limitations of current detection methods, such as Amplex Red and PO1, which lack sensitivity and selectivity at low levels [54]. Additionally, quantifying H_2_O_2_ levels in these cells would reflect the overall concentration within a heterogeneous cell population, where individual cells express varying levels of H_2_O_2_ due to differences in viral integration copy numbers within the genome. Given this variability, it may be more meaningful to measure H_2_O_2_ concentrations in cells expressing different levels of GFP (high, moderate, or low) to assess their impact on cellular homeostasis. However, implementing such an approach entails significant complexity and difficulty in both the experimental setup and subsequent data interpretation. Challenges encompass defining the thresholds for mild, low, or moderate levels of GFP expression, a task complicated by variations between different cell types and different fluorophores, as well as by the diverse subcellular localization of GFP. Additionally, the complexity of such studies is increased by the significance of the rate of H_2_O_2_ production over time rather than its concentration at a specific time point. This temporal aspect is critical because the fluorescence intensity of a given fluorophore does not linearly correlate with its expression levels, primarily due to variations in protein folding, chromophore maturation, and degradation rates. Therefore, the half-time of chromophore maturation is an important parameter to consider, along with the concentration and brightness of that fluorophore. Moreover, the HyPer7 biosensor itself can be viewed as a consumer of H_2_O_2_, as each detected H_2_O_2_ is reduced into water by HyPer7. This intricate interplay underscores the complexity inherent in accurately interpreting H_2_O_2_ dynamics within cells as a direct effect of HyPer7 expression.

In this study, we present insights into the impact of HyPer7 biosensor expression, particularly in association with its commonly utilized method of stable cell line generation via viral transduction. Therefore, we emphasize the necessity of implementing proper controls when using HyPer7 in conventional methods, such as validating results with non-fluorophore-based H₂O₂ probes and analyzing related genes like NRF2 and its targets to better understand the oxidative stress responses. Based on our findings, we strongly recommend utilizing HyPer7 sensors under 5 kPa O₂ conditions to ensure more reliable and accurate results. Additionally, we acknowledge the potential implications of HyPer7 overexpression on study outcomes. Nonetheless, our results highlight the limitations of common biosensor usage methods and the need for a thoughtful reassessment. Utilizing genome editing tools targeted at genomically safe harboring regions appears to be the most prudent strategy, enabling precise editing with minimal disruption at gene coding loci and providing control over copy number variations [55,56]. Additionally, fine tuning biosensor expression levels using truncated versions of low-strength CMV promoters could be an alternative strategy [57]. However, the development of brighter fluorescent proteins [[58], [59], [60]] will open up the possibility of performing experiments with lower expression levels of a given biosensor, sufficient to detect a workable signal-to-noise ratio and discard potential cell homeostasis perturbations. Additionally, our findings establish a connection with pathways related to oxidative stress, offering valuable insights for HyPer7 users that have not been previously explored.

## CRediT authorship contribution statement

**Sarah Barakat:** Writing – review & editing, Writing – original draft, Visualization, Methodology, Investigation, Formal analysis, Conceptualization. **Şeyma Çimen:** Methodology, Formal analysis. **Seyed Mohammad Miri:** Formal analysis. **Emre Vatandaşlar:** Formal analysis. **Hayriye Ecem Yelkenci:** Formal analysis. **Alejandro San Martín:** Writing – original draft, Validation, Supervision. **Mustafa Çağlar Beker:** Supervision, Formal analysis. **Kıvanç Kök:** Writing – review & editing, Writing – original draft, Supervision, Investigation, Formal analysis, Conceptualization. **Gürkan Öztürk:** Writing – review & editing, Resources. **Emrah Eroglu:** Writing – review & editing, Writing – original draft, Supervision, Investigation, Funding acquisition, Conceptualization.

## Competing interests

The authors declare that they have no competing interests.

## Declaration of use of generative AI and AI-assisted technologies in the writing process

During the preparation of this work, the authors utilized ChatGPT to improve the language. After employing this tool, the authors thoroughly reviewed and edited the content as necessary and take full responsibility for the content of this publication.

## Declaration of competing interest

None.

## Data Availability

Data will be made available on request.
