# Supplementary material for: Bioenergetic shift and proteomic signature induced by lentiviral-transduction of GFP-based biosensors
Source: Redox Biol. 2024 Nov 2;78:103416. doi: 10.1016/j.redox.2024.103416 (PMC11574814; doi:10.1016/j.redox.2024.103416)
Supplement: Multimedia component 1 [file mmc1.docx]

**10. Supplementary**

**
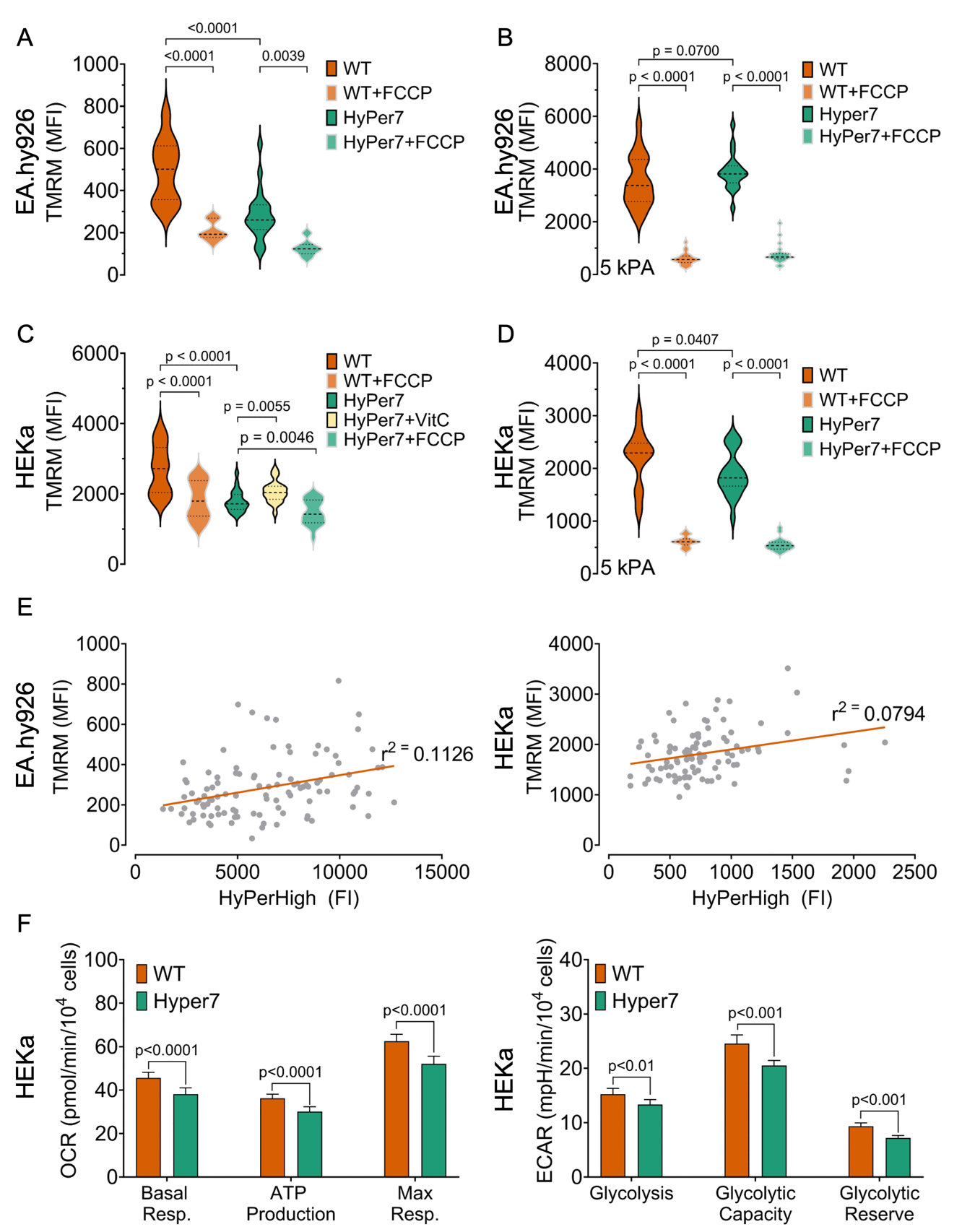
Supp. Fig. S1**

**Supp. Fig. S1:** **A, B)** Violin plots showing statistical analysis of the mean fluorescence intensity (MFI) of 20 nM TMRM stained HyPer7-expressing EA.hy926 cells (green) in comparison to wild-type cells (orange), with or without FCCP treatment (light colors), for cells cultured at 18 kPa (A), or 5 kPa (B). **C,D)** Violin plots showing statistical analysis of the mean fluorescence intensity (MFI) of 20 nM TMRM stained HyPer7-expressing HEKa cells (green) in comparison to wild-type cells (orange), with or without FCCP treatment (light colors), and treated with vitamin C (yellow), adapted to 18 kPa O_2_ (C) or 5 kPa O_2_ (D), Analysis reported as mean ± s.e.m. **E)** Correlation analysis of Hyper7 expression level to TMRM staining levels in EA.hy926 cells (left panel) and HEKa cells (right panel). **F)** Bar graphs showing comparison of OCR (left panel**)** and ECAR of HyPer7-expressing HEKa cells (green) in comparison to wild-type cells (orange). The data are representative of two independent experiments with n = 3 for each cell line. Data are expressed as mean ± s.e.m.

**
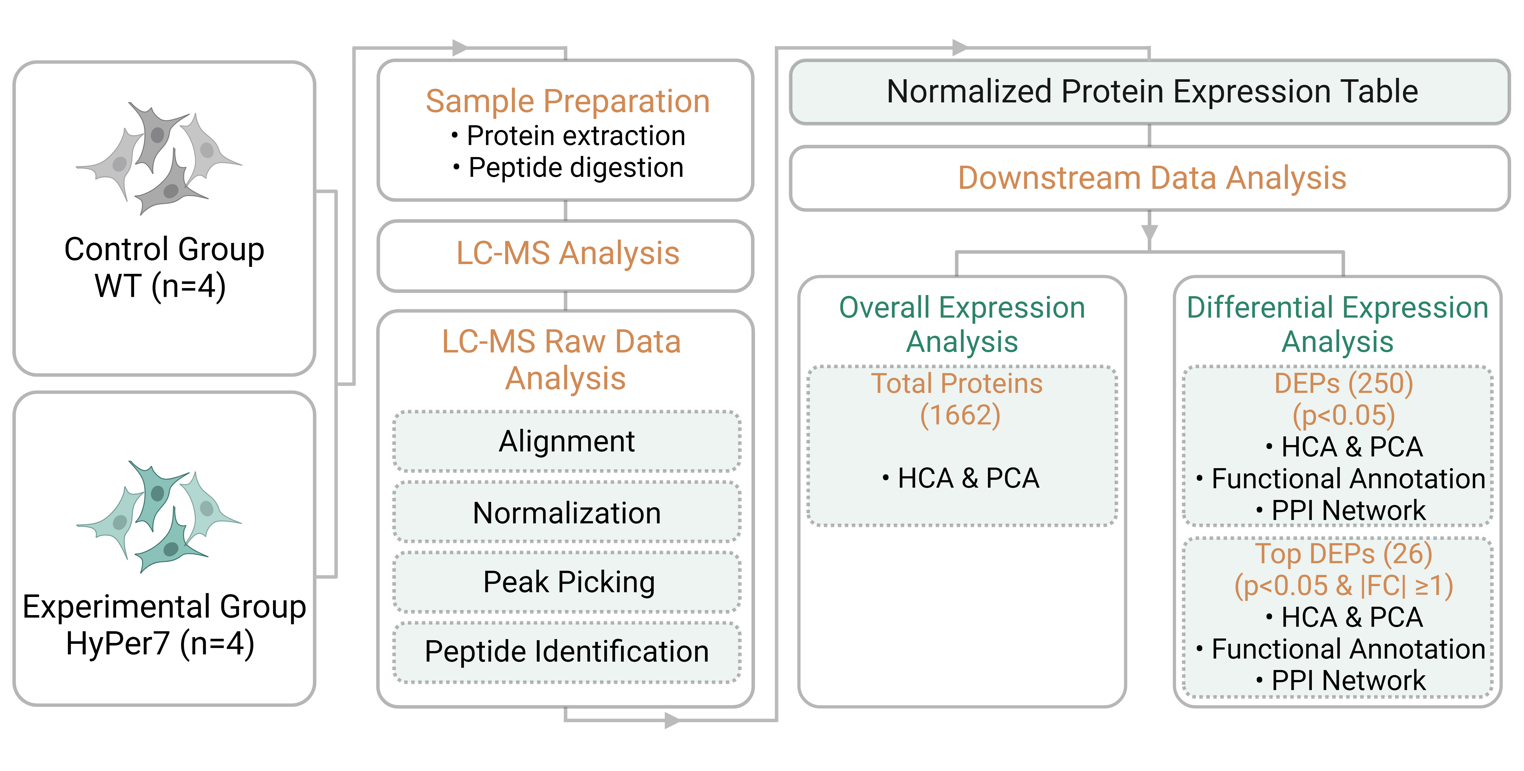
Supp. Fig. S2**

**Supp. Fig. S2:** Proteomics experiment and data analysis workflow: untargeted comparative proteomics analysis was performed on EA.hy926 endothelial cells; wild-type and cells expressing the cytosolic HyPer7 biosensor.

**Supp. Fi g. S3**

**
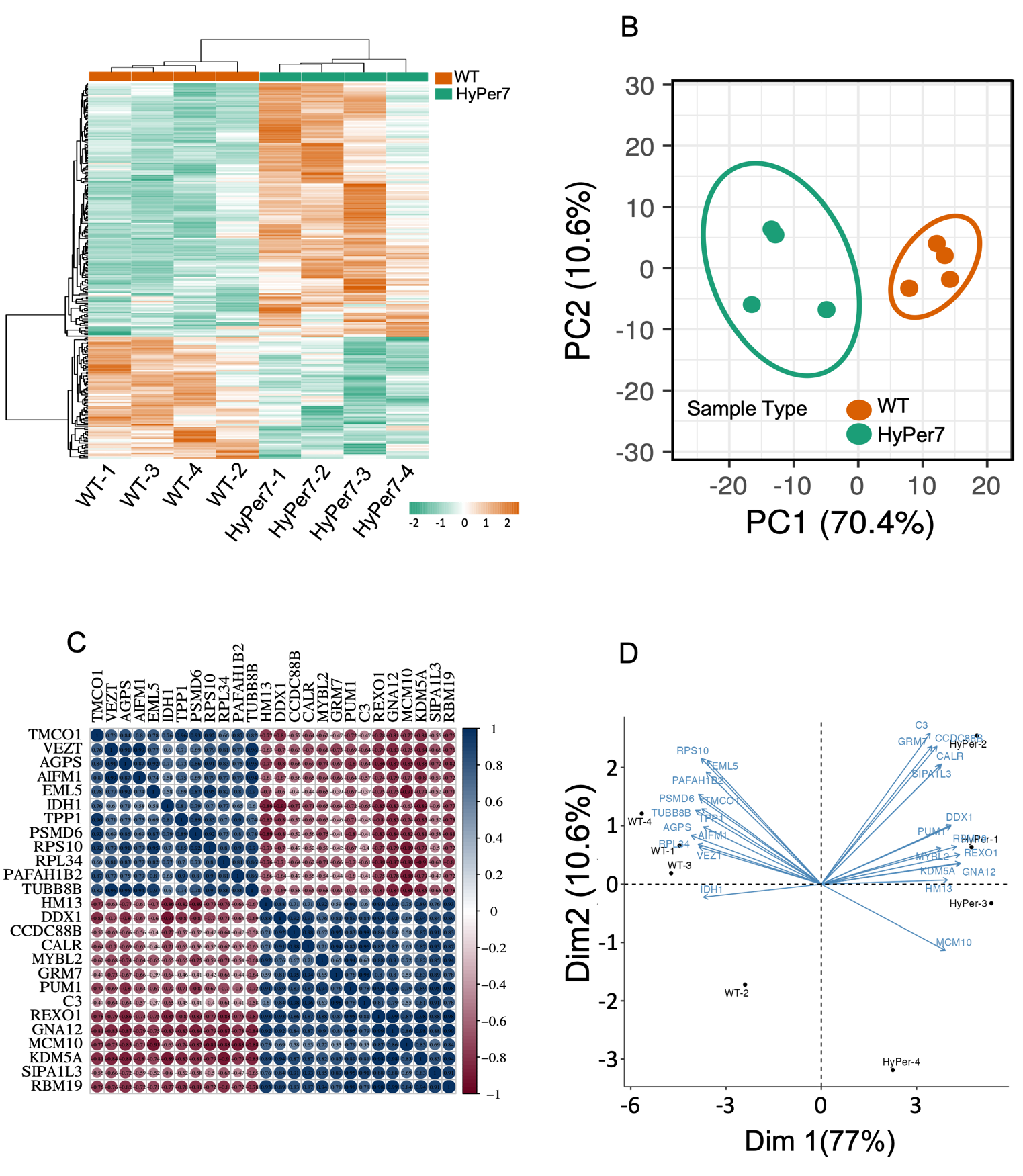
Supp. Fig. S3:** **A)** Hierarchical clustering heatmap analysis for DEPs. The profile highlights the clustering of samples according to their sample type: HyPer7-expressing cells compared to the control wild-type. The scale of orange and green color shows upregulated and downregulated protein expression, respectively. Scaling was applied to rows, cluster orientation was bidirectional, Pearson correlation was used for the distance metric, and average linkage was applied as the linkage method. **B)** PCA for DEPs. Graphs display the separation of the two sample groups, HyPer7 (green) compared to control wild-type cells (orange), based on the protein’s expression. The X and Y axes show principal component 1 and principal component 2, respectively. Prediction ellipses are such that, with a probability of 0.95, a new observation from the same group will fall inside the ellipse. **C)** Correlation Coefficient Plot reporting Pearson correlation coefficients of the top DEPs. The bar on the left side of the map indicates the color legend of the Pearson correlation coefficients. **D)** Principal Component Analysis (PCA) Biplot of top DEPs illustrating the distribution of wild-type and HyPer7 samples across the multidimensional space defined by the first two principal components (Dim1, Dim2). Samples are denoted in black, with distances and angles between samples reflecting the relationships and variations in the samples. The vectors indicate the contribution and directionality of each protein in shaping the principal components.

**Supp. Fig. S4**

**Supp. Fig. S4: A)** Functional enrichment analysis of top DEPs: Dot plot of GOTERM enriched terms related to cellular components (GO-CC), and molecular functions (GO-MF). **B)** Functional enrichment analysis of the subset of the reconstructed DEPs network identified in relation to the term “cellular responses to oxidative stress”. Dot plot of enriched functional annotation terms related to biological processes (GO-BP), molecular functions (GO-MF), REACTOME, and WikiPathway databases. The circle size represents the number of proteins enriched in each term, and the circle color represents the significance of enrichment.

**
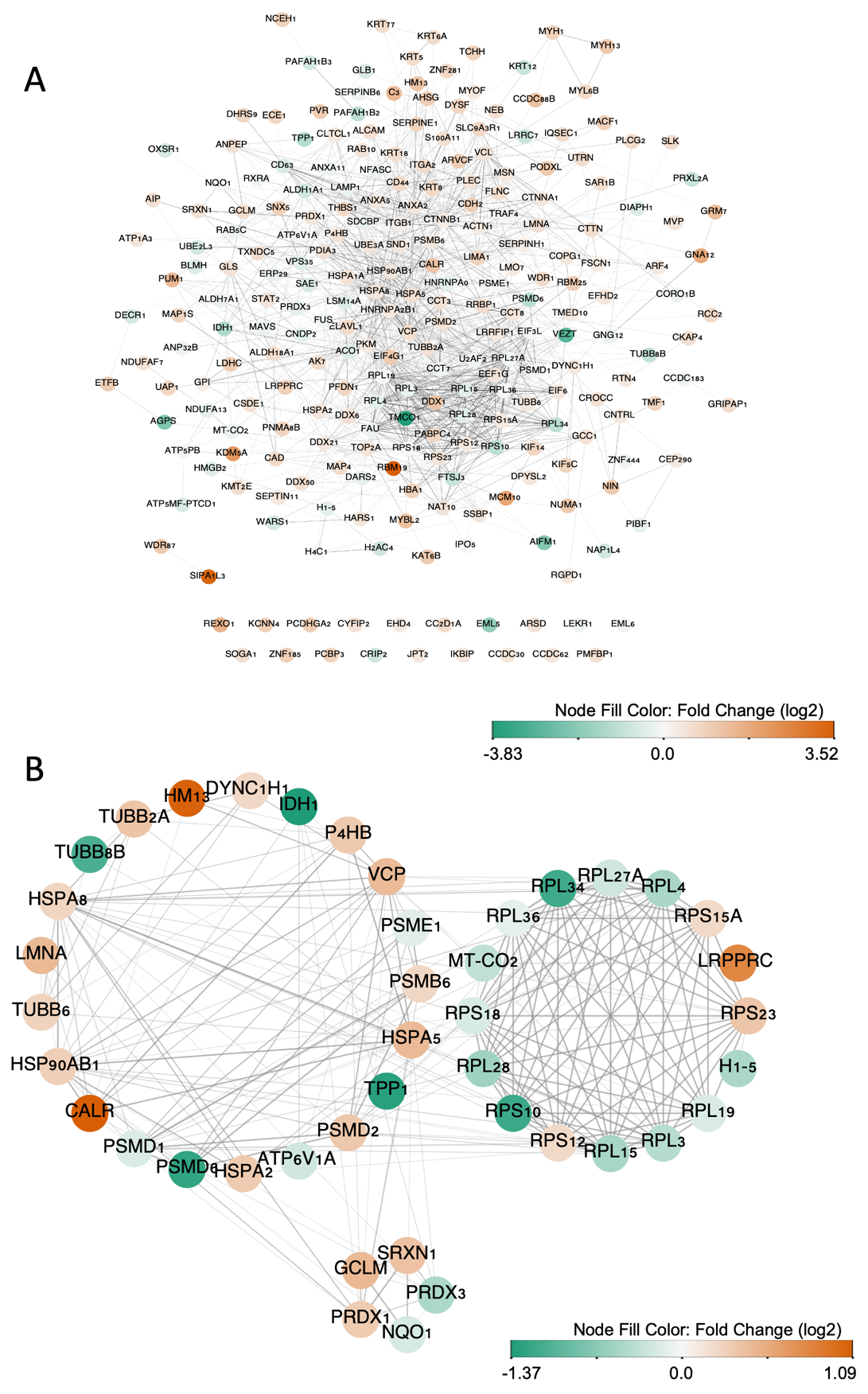
Supp. Fig. S5**

**Supp. Fig.S5:** **A)** Protein-protein interaction network analysis of identified DEPs. **B)** Protein-protein interaction network analysis of the subset of DEPs identified in relation to the term “cellular responses to stress”. Clustering was performed using the k-means clustering method. Nodes represent proteins, and edges represent PPI (retrieved from the String database). The color bar shows log2 (fold change). Hereby, up-regulation is shown in (orange), and down-regulation in (green). String was used for data network reconstruction, functional annotation, and clustering. Cytoscape was used for visualization.**Supp. Table S1:** List of identified top DEPs in HyPer7-expressing cells compared to wild-type. The symbol and the name of the identified proteins, along with the log2 fold change, and p-values, are shown for each protein. Additionally, the proteins that were identified as associated with “NRF2 transcription factor”, or “cellular responses to stress” based on functional annotation analysis are shown in the last column.

| # | Identified  Protein | Protein Name | Log2 (Fold Change) | P-value | Notes |
| --- | --- | --- | --- | --- | --- |
| 1 | KDM5A | Lysine demethylase 5A | 2.148 | 1.61E-05 | NRF2 |
| 2 | REXO1 | RNA exonuclease 1 homolog | 1.497 | 1.14E-04 |  |
| 3 | GNA12 | G protein subunit alpha 12 | 1.755 | 1.40E-04 | NRF2 |
| 4 | RPL34 | Ribosomal protein L34 | -1.159 | 3.14E-04 | NRF2 & Cellular responses to stress |
| 5 | RBM19 | RNA binding motif protein 19 | 3.515 | 1.46E-03 |  |
| 6 | MCM10 | Minichromosome maintenance 10 replication initiation factor | 1.821 | 3.92E-03 |  |
| 7 | HM13 | Histocompatibility minor 13 | 1.055 | 3.95E-03 | NRF2 & Cellular responses to stress |
| 8 | IDH1 | Isocitrate dehydrogenase (NADP(+)) 1 | -1.366 | 4.13E-03 | Cellular responses to stress |
| 9 | DDX1 | DEAD-box helicase 1 | 1.071 | 4.87E-03 | NRF2 |
| 10 | PAFAH1B2 | Platelet activating factor acetylhydrolase 1b catalytic subunit 2 | -1.153 | 6.55E-03 | NRF2 |
| 11 | TUBB8B | Tubulin beta 8B | -1.091 | 6.83E-03 |  |
| 12 | AGPS | Alkylglycerone phosphate synthase | -2.186 | 7.25E-03 | NRF2 |
| 13 | TPP1 | Tripeptidyl peptidase 1 | -1.302 | 7.34E-03 | NRF2 & Cellular responses to stress |
| 14 | PUM1 | Pumilio RNA binding family member 1 | 1.328 | 1.06E-02 |  |
| 15 | RPS10 | Ribosomal protein S10 | -1.178 | 1.08E-02 | Cellular responses to stress |
| 16 | PSMD6 | Proteasome 26S subunit, non-ATPase 6 | -1.245 | 1.16E-02 | NRF2 & Cellular responses to stress |
| 17 | VEZT | Vezatin, adherens junctions transmembrane protein | -2.872 | 1.21E-02 | NRF2 |
| 18 | TMCO1 | Transmembrane and coiled-coil domains 1 | -3.832 | 1.58E-02 | NRF2 |
| 19 | MYBL2 | MYB proto-oncogene like 2 | 1.146 | 1.91E-02 |  |
| 20 | AIFM1 | Apoptosis inducing factor mitochondria associated 1 | -2.128 | 1.92E-02 | NRF2 |
| 21 | CALR | Calreticulin | 1.086 | 2.16E-02 | NRF2 & Cellular responses to stress |
| 22 | EML5 | EMAP like 5 | -1.888 | 2.28E-02 | NRF2 |
| 23 | CCDC88B | Coiled-coil domain containing 88B | 1.074 | 2.35E-02 |  |
| 24 | SIPA1L3 | Signal induced proliferation associated 1 like 3 | 3.355 | 3.49E-02 |  |
| 25 | GRM7 | Glutamate metabotropic receptor 7 | 1.231 | 3.70E-02 | NRF2 |
| 26 | C3 | Complement C3 | 1.350 | 4.59E-02 |  |

**Supp. Table S2:** List of significantly enriched terms associated with the identified top DEPs in HyPer7- expressing cells compared to wild-type. The name of the enriched term, enrichment value, p-value of the enrichment test, and the count of top DEPs associated with each term, together with the annotation database, are shown.

| Term | Enrichment | P-value | Count | Database |
| --- | --- | --- | --- | --- |
| Cellular responses to stress | 2.3 | 5.30E-02 | 7 | REACTOME_PATHWAY |
| Cellular responses to stimuli | 2.3 | 5.40E-02 | 7 | REACTOME_PATHWAY |
| GPCR downstream signaling | 6.6 | 6.70E-02 | 3 | REACTOME_PATHWAY |
| Signaling by GPCR | 6.4 | 7.00E-02 | 3 | REACTOME_PATHWAY |
| GPCR ligand binding | 21.2 | 8.60E-02 | 2 | REACTOME_PATHWAY |
| Peroxisomal protein import | 21.2 | 8.60E-02 | 2 | REACTOME_PATHWAY |
| NRF2 | 1.8 | 7.70E-03 | 15 | UCSC_TFBS |
| BACH2 | 1.5 | 2.00E-02 | 17 | UCSC_TFBS |
| TAL1BETAITF2 | 1.4 | 3.00E-02 | 18 | UCSC_TFBS |
| HSF1 | 1.7 | 4.10E-02 | 13 | UCSC_TFBS |
| HOX13 | 1.4 | 5.30E-02 | 16 | UCSC_TFBS |
| ZID | 1.4 | 5.70E-02 | 17 | UCSC_TFBS |
| LMO2COM | 1.3 | 6.50E-02 | 19 | UCSC_TFBS |
| MSX1 | 1.5 | 6.60E-02 | 15 | UCSC_TFBS |
| OCT | 1.4 | 7.10E-02 | 16 | UCSC_TFBS |
| secretory granule lumen | 4.6 | 4.80E-02 | 4 | GO-CC |
| nucleolus | 2.4 | 4.90E-02 | 7 | GO-CC |
| lumenal side of endoplasmic reticulum membrane | 20.7 | 9.00E-02 | 2 | GO-CC |
| [Exonuclease activity](http://www.ebi.ac.uk/QuickGO/GTerm?id=GO:0004527) | 24.6 | 7.60E-02 | 2 | GO-MF |
